# Endometrial injury concurrent with hysteroscopy increases the expression of Leukaemia inhibitory factor: a preliminary study

**DOI:** 10.1186/s12958-021-00877-z

**Published:** 2022-01-10

**Authors:** Suat Suphan Ersahin, Aynur Ersahin

**Affiliations:** 1 Altınbas University Department of Obstetrics and Gynecology, Istanbul, Turkey; 2 BAU Medical School Department of Obstetrics and Gynecology, IVF-Center, Istanbul, Turkey

**Keywords:** Endometrial injury, Hysteroscopy, Monopolar forceps, LIF mRNA, RT–PCR

## Abstract

**Objective:**

It is not known by which mechanism endometrial injury increases pregnancy rates. Leukaemia inhibitory factor (LIF) is a cytokine involved in wound healing and implantation. The aim of this study was to determine the change in endometrial LIF mRNA expression before and after mechanical injury during hysteroscopy.

**Methods:**

Forty patients with a history of two or more unsuccessful implantations who decided to undergo hysteroscopy in the proliferative phase were divided into two equal groups: one with endometrial injury (scratching group) and the other with noninjury (control group). Endometrial sampling was conducted before injury on the patients in the scratching group, and then injury was performed with monopolar needle forceps. Only diagnostic hysteroscopy was performed on the patients in the control group. Endometrial tissues were collected using a Pipelle catheter between Days 20 and 23 of the mid-luteal phase of the next cycles in both the scratching and control groups. Endometrial LIF mRNA expression was evaluated with the use of reverse-transcription polymerase chain reactions.

**Results:**

Relative changes in mRNA expression levels of the LIF gene in endometrial samples taken before and after injury were calculated using the 2-ΔΔCt method, and the fold changes obtained were compared between and within the groups. Compared with preinjury values, an 11.1-fold increase was found in postinjury LIF mRNA expression in patients with monopolar forceps injury (*p* < 0.001). There was a 3.9-fold significant increase in postinjury LIF mRNA levels compared with those in the control group (*p* < 0.02).

**Conclusions:**

The fertility-promoting effect of hysteroscopy-guided mechanical endometrial injury may be mediated by LIF mRNA.

## Introduction

Implantation failure is a rate-limiting step in patients using assisted reproductive technology. In terms of both the patient and the physician, the number of interventions after this stage is very limited. Mechanical endometrial injury comes to the aid of the physician. It has been reported that pregnancy rates have increased nearly twofold with therapeutic endometrial scratching [1, 2]. The procedure is minimally invasive and can be performed with a Pipelle cannula, a Novak curette, or hysteroscopy [1, 3–5]. The preferred application is mostly in the form of controlled damage to the entire endometrial cavity with a Pipelle cannula without anaesthesia. No complications other than mild pain have been reported. While implantation rates increase in caesarean scar tissue, uncontrolled and sharp curettages may lead to adhesion and infertility. For this reason, the necessary care should be shown while performing mechanical injury.

Although endometrial injury was reported to increase clinical pregnancy and live birth rates in patients with recurrent implantation failure [6], endometrial injury in the unselected patient group did not always yield the expected positive results [7, 8]. Hence, data on endometrial injury are not clear enough to suggest routine use of this method. The main reason for this is the lack of clear data on which patient group, when, how many times and which instruments should be used [9]. The limited data on the mechanism of action through which injury increases pregnancy rates are also an important limitation [7, 9]. Although the number of studies on the contribution of local injury to pregnancy rates is very large, the mechanism by which injury increases pregnancy rates is not clearly known [7–9]. It has been suggested that injury contributes to pregnancy rates by triggering the synthesis of inflammatory molecules, increasing the number of uterine natural killer cells or increasing the expression of some receptivity genes [10–12]. However, there is no clinical study showing the net effect of mechanical damage on the endometrium.

Leukaemia inhibitory factor (LIF) is a cytokine involved in both wound healing and implantation [13–15]. This cytokine belongs to the IL-6 family and plays an important role in embryo-endometrium interactions [16]. Following binding to its specific receptor (gp130), LIF activates signal transducers and activators of the transcription 3 signalling pathway and regulates proliferation, differentiation, and cell survival in the endometrium [17, 18]. Failed expression of endometrial LIF may cause infertility in animals [19]. The regulatory effect of LIF on thymic T cell maturation contributes to the prevention of foetal rejection [20]. In addition to its well-known effects on implantation, LIF is a pleotropic cytokine that is also involved in injury-induced wound healing by regulating cell growth and differentiation [13]. The LIF pathway is also disrupted in the endometrium of patients with unexplained infertility, particularly those with endometriosis and endometrioma [21, 22]. Consistent with this finding, Margioula et al. [23] showed impaired LIF expression levels only in women with unexplained infertility, while LIF-R expression was impaired in women with endometriosis or poor responders [24]. They also reported that endometrial expression of LIF and LIF R is significantly reduced in the epithelial cells of infertile women [24]. Because endometrial injury induces a type of wound healing process, this study was designed to determine the effects of hysteroscopic mechanical endometrial injury on LIF mRNA expression in patients with a history of failed implantation. The expression levels of LIF mRNA were compared in endometrial samples collected before and after endometrial injury.

## Materials and methods

Fifty infertile patients with a history of two or more failed implantations who decided to temporarily interrupt the treatment were included in the study. Hysteroscopic decisions were made in all cases to exclude possible endometrial causes underlying the failed implantation. The subjects were recruited from a university-based infertility clinic between January 2019 and June 2021. Approval for the study was obtained from the Institutional Review Board. Hysteroscopy was performed between Days 10 and 12 of the proliferative phase. Ten patients who were found to have polyps, fibroids, adhesions or a uterine septum in the cavity during hysteroscopic observation and who underwent surgical procedures were not included in the study. Forty patients with a normal endometrial cavity were divided into two groups, 20 patients in each group: one with endometrial injury (scratching group) and the other with non-injury (control group). The patients in the control group were selected from those who did not accept the mechanical injury procedure. Risk factors for recurrent implantation failure, including advanced maternal age, smoking status, body mass index, presence of specific autoantibodies, thrombophilia panel results, infectious organisms, and uterine pathologies such as polyps and myomas as well as congenital anomalies were investigated. Sperm analysis was also performed to exclude male-related causes.

For LIF analysis, endometrial sampling was first performed, and then mechanical endometrial injury was performed in the same cycle while the patient was under anaesthesia. Endometrial sampling was performed before mechanical endometrial injury from the patients in the scratching group, and then local endometrial injury was performed with monopolar electric energy with needle forceps. Only diagnostic hysteroscopy was performed on the patients in the control group. Endometrial sampling was not performed due to the possibility of increasing LIF expression in the control group. Endometrial tissues were collected using a Pipelle catheter between Days 20 and 23 of the mid-luteal phase of the next cycles of patients in both the scratching and control groups. The endometrial samples were washed three times with a sterile saline solution to remove blood, transferred into RNA stabilization buffer, and stored at − 20 °C for later analysis. Participants were asked to use one of the nonhormonal methods of contraception during the study. Women with endometrioma or hydrosalpinx thought to affect implantation were not included in the study. Patients over 37 years of age and those with a poor response to ovarian stimulation were also excluded from the study. Positive pregnancy test results and clinical pregnancy rates of the groups with and without scratching were calculated. Clinical pregnancy was defined as evidence of a gestational sac, confirmed by ultrasound examination at the 4th week of transfer.

### Expression analysis

For expression analysis of targeted genes, total RNA was extracted from homogenized tissue material obtained from endometrium using the GeneAll Hybrid–RTM RNA Kit (GeneAll, Korea). After isolation of total RNA, 1 μg of extracted RNA was used for cDNA synthesis, which was performed with a GeneAll 2X HyperScriptTM One-Step cDNA Kit (GeneAll, Korea) in accordance with the manufacturer’s instructions. The expression levels of LIF mRNA were detected by using SYBR Green methodology (Eva Green PCR Master Mix, Biotium) in the Step-One Plus Real Time PCR System (Applied Biosystems, USA) with 25 μl of total reaction volume. The housekeepingGAPDH gene was used for normalization. The mRNA levels were calculated using the comparative ΔCt method (Ct of target gene - Ct of reference gene). Relative changes in the mRNA expression levels of the analysed genes were calculated using the 2^-ΔΔCt^ method (ΔCT treated - ΔCT untreated), and the obtained 2^-ΔΔCt^data were used for statistical analysis [25].

### Statistical analysis

Data wereanalysed with theStatistical Package for Social Sciences software 21.0 for Windows package software (SPSS). The normality distribution of the data was tested with the Kolmogorov–Smirnov test. Continuous variables were analysed by means of analysis of variance with the post hoc Tukey procedure and Mann–Whitney U test. Categorical data were analysed by means of the Pearson chi-square test. Data are presented as the means ± SD. A *P* value of <.05 was considered statistically significant. Fold increases were considered to be positive for transcript overexpression when the correspondingmRNA level was ≥3-fold higher than that of initial transcript expression and negative if < 2-fold.

## Results

The demographic and clinical characteristics of each group of participants are shown in Table 1. Age, BMI, endometrial thickness, infertility duration, and the number of unsuccessful embryo transfers of patients in both groups were found to be similar. Hysteroscopy was performed in all cases under optimum conditions. Cases that turned into interventional hysteroscopy due to an endometrial pathology detected by diagnostic hysteroscopy were excluded from the study. In the injury group, 8 out of 20 cases had a positive pregnancy test (40%). The pregnancy test was positive in 6 out of 20 cases in the group that did not undergo injury (30%). The positive pregnancy test rates were significantly higher in the injury groupthan in the control group (*p* < 0.03). Clinical pregnancy was detected in 7 cases (35%) in the injury group and 5 cases (25%) in the control group, and this difference was recorded as significant (*p* < 0.02).

**Table 1:** Demographic characteristics of scratching and non-scratching groups

	Scratching (***n*** = 20)	Non-scratching (***n*** = 20)	P*
**Age (y)**	**29.2 ± 1.02**	**28.7 ± 0.33**	**0.30**
**BMI (kg/m2)**	**24.8 ± 1.92**	**25.1 ± 0.34**	**0.06**
**Infertility duration (y)**	**5.21 ± 1.40**	**4.79 ± 1.10**	**0.08**
The number of failed IVF-ET attempt	**3.4 ± 1.2**	**3.2 ± 2.08**	**0.09**
Endometrial thickness on hysterosocpy day (mm)	10.5 ± 3.20	9.77 ± 2.06	0.70
FSH (mIU/mL)	**4.98 ± 2.02**	**5.02 ± 1.66**	0.55
LH (mIU/mL)	**7.66 ± 2.11**	**8.30 ± 2.05**	0.07
**Testosterone (ng/mL)**	**0.55 ± 1.55**	**0.49 ± 0.22**	**0.65**
**Glucose (mg/dL)**	**85.7 ± 6.54**	**80.4 ± 3.05**	**0.50**
Positive beta hCG test (%)	8 (40%)	6 (30%)	0.03
Clinical pregnancy (%)	7 (35%)	5 (25%)	0.02
LIF mRNA	11.1-fold increase after injury (*p* < 0.001) 3.9 times increase compared to the control group (*p* < 0.02)		

Data presented as means ± SD. BMI; body mass index, *p < 0.05

Relative changes in mRNA expression levels of the LIF gene in endometrial samples taken before and after injury were calculated using the 2^-ΔΔCt^ method, and the fold changes obtained were compared between and within the groups. First, the mean ΔCt values ​​of the patients in the scratching group were determined before and after the injury. As a result of the calculations made according to ΔCt and 2^-ΔΔCt^ values, the change detected due to injury corresponded to an approximately 11.1-fold increase in LIF mRNA expression. The up regulation of LIF mRNA expression after injury was statistically significant (*p* < 0.001) compared with the preinjury values. Since the fold increase in LIF mRNA expression was > 3, it was accepted as up regulation. As a result of the calculations made with the ΔCt and 2^-ΔΔCt^ values ​​obtained from the endometrial samples made in the mid-luteal phase in the control group patients, there was an approximately 3.9-fold increase in LIF mRNA expression in the injury group compared to the control group, and this increase was statistically significant (*p* < 0.02). The post-injury LIF mRNA expression levels of the patients who underwent hysteroscopic injury were found to be significantly higher than those of the control group patients who underwent diagnostic hysteroscopy but did not undergo injury. The mean values of LIF mRNA by real-time PCR in the scratching and control groups were 25 and 29 arbitrary units (AU), respectively.

## Discussion

Although the effect of mechanical endometrial injury on implantation and pregnancy rates is variable, it has become an almost routine practice for many clinicians to perform endometrial scratching in patients with implantation failure, as most studies report that it increases pregnancy rates [9]. On the other hand, although there are clinical data that endometrial injury is beneficial for the success of achieving pregnancy in patients with recurrent implantation failure, this does not mean that endometrial scratching should be offered to everyone in daily practice [7–9]. Indeed, we do not have enough scientific data to recommend endometrial injuryto increase pregnancy rates in any patient group [7, 8]. While almost all of the studies were looking for answers to the questions of which patient group and when to induce the injury, which instrument should we use for the injury, and how many times we should perform the injury, the physiological mechanisms underlying the success of the method were ignored [9]. The following mechanisms have been suggested in a small number of studies on the mechanism by which injury increases pregnancy rates: (i) by increasing the inflammatory response in the endometrium [12], (ii) by increasing the synthesis of growth factors that promote wound healing [1], (iii) by promoting decidualization [11], and by increasing the expression of some endometrial genes [10].

Many endometrial injury studies have suggested investigating the expression of receptivity genes to elucidate the possible biological mechanism, but such a study has not been conducted thus far [7, 9]. Leukaemia inhibitory factor (LIF) is a cytokine responsible for embryo implantation and subsequent embryonic stem cell differentiation [26]. LIF has many physiological functions apart from its role in implantation. It is one of the main proinflammatory cytokines secreted in response to injury and is responsible for wound healing [13].. Since the physiological process that takes place in the endometrium after mechanical endometrial injury is a kind of wound healing, we investigated the change in LIF mRNA levels before and after injury in patients with a history of implantation failure. There was an approximately 11-fold increase in the LIF mRNA levels measured after the injury compared to the expression levels measured in the endometrium samples taken before the injury. Similarly, when compared to the patients in the control group who were not injured, an approximately fourfold increase was found in the LIF mRNA expression levels measured in the patient group exposed to injury. Barash et al. suggested that the changes that occur in the endometrium after injury are a type of wound healing and that cytokines and growth factors released in response to injury both heal the endometrium and contribute to implantation [1]. The reason for this increase in LIF release in the postinjury period may be the functional and biological similarity of LIF to many cytokines involved in implantation and wound healing and its attachment to the same receptor. LIF has multiple functions, as it stimulates the JAK-STAT pathway by binding to the gp130 receptor together with many cytokines, such as interleukin-6, interleukin-11, ciliary neurotrophic factor, and oncostatin M [27]. Increasing LIF after endometrial injury increases both wound healing and implantation rates by showing the effect of all these cytokines alone.

Defects in endometrial LIF expression have been reported in many infertile patient groups. There is a decrease in LIF expression in the endometrium of both hydrosalpinx patients and endometriosis patients [28, 29]. An 8–10-fold increase in LIF levels has been reported after salpingectomy [28]. Similarly, in a study by our team, we reported decreased endometrial LIF mRNA expression in endometrioma patients and an approximately 1.8-fold increase in LIF levels after endometrioma resection [15]. All these studies show that LIF expression is affected by the underlying disease and that its expression is normalized following treatment of the existing pathological condition. The endometrium of patients with implantation failure was seen as normal in both ultrasonography and hysteroscopic examination. However, an endometrium with a normal appearance and sufficient thickness may not be normal at the molecular and histological levels [30]. For this reason, the intense increase in LIF mRNA levels after injury indicates the existence of a cellular problem in the endometrium of these patients. In good agreement with this finding, defective LIF expression in the endometrium may impair the release of type 2 T helper cytokines, leading to recurrent implantation failure [31]. Insufficient LIF levels in the endometrium of patients with endometrioma or hydrosalpinx suggest that the expression of this cytokine is also insufficient or defective in cases of implantation failure [15, 28]. A 4-fold increase in LIF in the postinjury group compared to the non-injured group supports this idea. Injury may cause temporary recovery of pathological changes in the endometrium and increase LIF secretion. The fact that LIF cDNA treatment is effective for skin allograft survival is important evidence that increased LIF mRNA levels may also be effective for recovery of implantation after endometrial injury [32]. This idea needs to be confirmed by further studies.

For a successful pregnancy, the balance between LIF-producing T cells and Th2-producing T cells must be maintained at the implantation site [31]. In connection with this status, increased LIF expression can increase the implantation rates by changing the Th1 and Th2 cytokine balance in favour of Th2 in endometrial cells. The 11-fold increase in LIF mRNA expression after endometrial injury may increase T cell maturation, leading to the emergence of Th2 dominance and prevention of foetal rejection [33]. The increase in implantation and pregnancy rates in the postinjury period suggests tolerance to embryo rejection. As there is a clear link between allograft tolerance and cytokine expression, there may be a link between implantation rates reported to be increased after injury and LIF expression [13, 32]. The embryo is a semiallograft in the mother and therefore is at risk of rejection during the implantation process. The intense increase in LIF mRNA expression after injury may increase implantation rates by regulating the number of immune cells that are responsible for embryo rejection and tolerance [34]. Hamelin-Morrissette et al. reported that LIF regulates the activationof inflammatory signals in macrophages and trophoblast cells [35]. However, since these cell types and amounts were not studied in our investigation, this interpretation should be confirmed with further studies.

There are some limitations of our study, especially with respect to the small number of participants. Two important handicaps are that we did not correlate the increase in mRNA expression with protein expression and that we did not localize the LIF expression immunohistochemically. Because of the selected patient group, our results are not applicable to patients not diagnosed with RIF. Another weakness is that only patients with hysteroscopic injury were included in the study, and there was no patient group with Pipelle-induced injury. In fact, we had a specific purpose in selecting the participants from among the patients who would undergo hysteroscopy. Compared to the injuries performed using a Pipelle cannula, the results of the injury studies performed with hysteroscopy are more homogeneous. Studies using a Novac curette or monopolar forceps in hysteroscopic injury have yielded similar results [3–5]. On the other hand, there are some negative reasons related to the method itself that cause the results to be different in studies using Pipelle catheters. The process is conducted without seeing the cavity, the method changes according to the person who does it, and it is not certain that the entire cavity is exposed to the injury. For these reasons, we chose the participants from among the patients who decided to undergo hysteroscopic injury in order to see the cavity and to createan injury in each patient in a standard way. Despite all these limitations, we have reported the effects of mechanical endometrial injury on endometrial LIF expression for the first time in this study.

Further research is necessary to clarify how and why LIF increases occur. In addition to studies investigating the possible changes in LIF and other receptivity genes after injury, there is a need for studies examining the restorative and implantation-promoting effects of LIF cDNA vector use in experimental injury models.

## Data Availability

No
